# Convenient, Rapid and Accurate Measurement of SVOC Emission Characteristics in Experimental Chambers

**DOI:** 10.1371/journal.pone.0072445

**Published:** 2013-08-28

**Authors:** Cong Liu, Zhe Liu, John C. Little, Yinping Zhang

**Affiliations:** 1 Department of Building Science, Tsinghua University, Beijing, China; 2 Department of Civil and Environmental Engineering, Virginia Tech, Blacksburg, Virginia, United States of America; The Ohio State University, United States of America

## Abstract

Chamber tests are usually used to determine the source characteristics of semi-volatile organic compounds (SVOCs) which are critical to quantify indoor exposure to SVOCs. In contrast to volatile organic compounds (VOCs), the sorption effect of SVOCs to chamber surfaces usually needs to be considered due to the much higher surface/air partition coefficients, resulting in a long time to reach steady state, frequently on the order of months, and complicating the mathematical analysis of the resulting data. A chamber test is also complicated if the material-phase concentration is not constant. This study shows how to design a chamber to overcome these limitations. A dimensionless mass transfer analysis is used to specify conditions for (1) neglecting the SVOC sorption effect to chamber surfaces, (2) neglecting the convective mass transfer resistance at sorption surfaces if the sorption effect cannot be neglected, and (3) regarding the material-phase concentration in the source as constant. Several practical and quantifiable ways to improve chamber design are proposed. The approach is illustrated by analyzing available data from three different chambers in terms of the accuracy with which the model parameters can be determined and the time needed to conduct the chamber test. The results should greatly facilitate the design of chambers to characterize SVOC emissions and the resulting exposure.

## Introduction

Semi-volatile organic compounds (SVOCs) are ubiquitous in indoor environments, with a significant number present as additives (e.g., plasticizers and flame retardants) in many indoor materials and products [1]–[4]. Due to their extremely low vapor pressure, SVOCs emitted from indoor sources readily partition to indoor media, including interior surfaces [5]–[6], airborne particles [7] and settled dust [8]. Human exposure to certain SVOCs is associated with adverse health effects, including asthma, allergies, bronchial obstruction [9]–[10], reproductive disorders [11]–[12] and endocrine disruption [13] and partitioning to indoor media plays an important role in determining the dominant route(s) of exposure [14].

To quantify indoor exposure to SVOCs in specific products, mass balance models are developed that predict indoor SVOC emissions, transport and intake [15]. Small chambers (e.g. Chamber for Laboratory Investigations of Materials, Pollution, and Air Quality (CLIMPAQ), Field and Laboratory Emission Cell (FLEC) and a sandwich-like chamber) are used to determine source parameters [16]–[18] and build confidence in the resulting models [19]. In contrast to VOCs [20]–[21], the sorption of SVOCs to chamber surfaces needs to be considered due to the much higher surface/air partition coefficients [16]. A strong sink effect results in a long time to reach steady state, frequently on the order of months, and complicates the mathematical analysis of the resulting data.

Depending on the design of the chamber, and the volatility of the SVOCs, there may be conditions for which the sink effect can be neglected. This would simplify the chamber experiments to determine the source characteristics in terms of experiment duration and mathematical analysis of data. If the sink effect must be included, neglecting the convective mass transfer resistance at sorption surfaces under suitable conditions can simplify the mathematical analysis of the experimental data. Finally, chamber studies can be greatly simplified in terms of both experimental duration and mathematical analysis if the material-phase SVOC concentration in the source can be regarded as constant. To improve the efficiency and effectiveness of chamber studies, the conditions under which these three assumptions (neglecting the sink effect, neglecting the convective mass transfer resistance at sorption surfaces, and regarding the material-phase SVOC concentration as constant) are valid need to be identified.

The objectives of this study are therefore to: (1) present a mass transfer analysis to describe the behavior of SVOCs in chambers and identify essential dimensionless parameters; and (2) determine the conditions for which the sink effect and convective mass transfer resistance at sorption surfaces may be neglected, and under which the material-phase SVOC concentration may be considered constant. The analysis is illustrated by analyzing available data from three very different chamber studies in terms of the accuracy with which the model parameters can be determined and the time needed to conduct the chamber test. The results can help quantify improvements in chamber design, facilitating the use of chambers to characterize SVOC emissions and exposure.

## Description of the Problem

**Figure 1:**
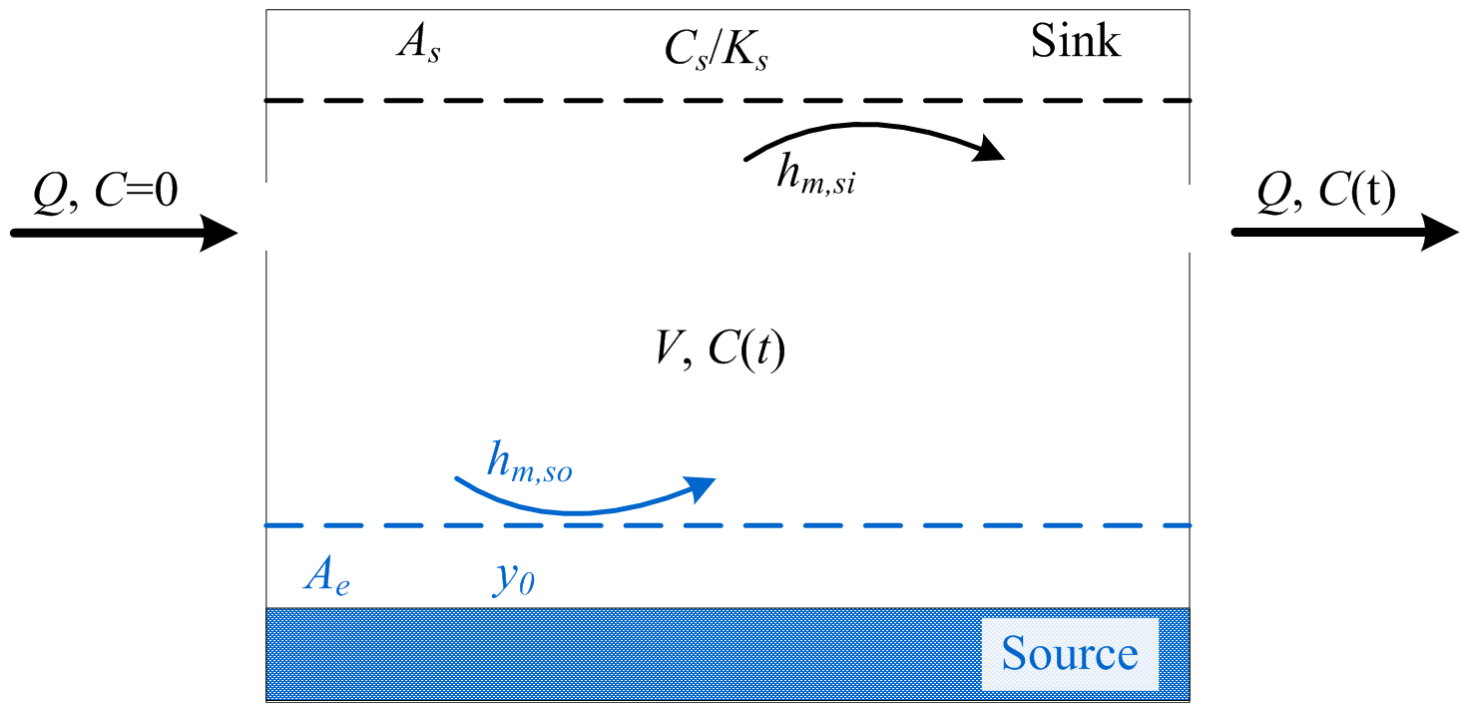
Schematic representation of SVOC source/sink behavior in a chamber.

Liu et al. [18] developed a model to describe emissions of SVOCs from a polymer slab in a chamber. The model is shown schematically in Figure 1.

The concentration of SVOCs used as additives in the source materials can usually be regarded as constant since they constitute 10–40 wt% of the source material [22]–[24]. SVOCs emit slowly from the source materials due to their high molecular weight and low vapor pressure [25]. The emission rate of an SVOC source in a chamber, *E* (µg/s), is given by:
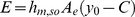
(1)

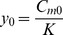
(2)


Similarly, the sorption rate at an interior sink surface, *S* (µg/s), is:
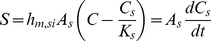
(3)and the gas phase SVOC concentration in a particle-free environment is given by:
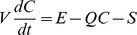
(4)where *h_m,so_* (m/s) is the mass transfer coefficient at the emission surface, *A_e_* (m^2^) the emission surface area, *y_0_* (µg/m^3^) the concentration of the SVOC in the air immediately adjacent to the surface, *C* (µg/m^3^) the gas-phase SVOC concentration in the chamber, *C_m0_* (µg/m^3^) the material-phase SVOC concentration, which is assumed to be constant, K a parameter describing the equilibrium between the material and the air, *h_m,si_* (m/s) the mass transfer coefficient at the sink surface, *A_s_* (m^2^) the sink surface area, *C_s_* (µg/m^2^) the SVOC concentration on the sink surface, *K_s_* (m) the SVOC partition coefficient between the sink surface and air, *V* (m^3^) the volume of air in the chamber, and *Q* (m^3^/s) the ventilation rate.

For chamber studies, the initial conditions are usually:
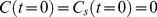
(5)


The analytical solution to equations (1)–(5) is:
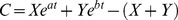
(6)where *X* and *Y* are given by:
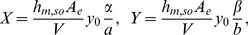
(7)and α and β are the roots of the following set of equations:
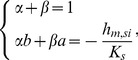
(8)with a and b given by the roots of the following equation:



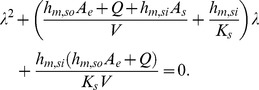
(9)When the sink effect is neglected, equation (3) simplifies to
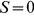
(10)


The analytical solution under these conditions is
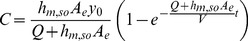
(11)


When convective mass transfer resistance at sorption surfaces is neglected, or *h_m,si_* becomes infinite, equation (3) simplifies to
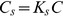
(12)


The analytical solution under these conditions is
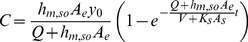
(13)


It should be noted that the model excludes airborne particles, a condition which can easily be achieved in laboratory experiments as clean air without particles is usually used. When assessing exposure to SVOCs in a realistic environment, airborne particles can play an important role [26] and the model needs to be adjusted to include them.

## Dimensionless Analysis

Dimensionless analysis of the source characteristics of VOCs in building materials was first completed by Xu and Zhang [21]. Using the dimensionless formulae, results obtained under one condition can be scaled to another condition, as long as the dimensionless parameters are the same. In addition, after being normalized and made dimensionless, the number of variables can be reduced. The dimensionless parameters for this study are defined as:
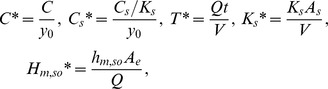


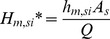



The dimensionless model development is presented in Supporting Information S1. Four key dimensionless parameters, instead of the original eight variables, can describe the SVOC concentration and the SVOC source/sink emission/sorption rate. The physical meaning of the four key dimensionless parameters is: T* is the dimensionless time, K_s_* is the dimensionless sorption capacity of sink surfaces, while H_m,so_* and H_m,si_* are the dimensionless mass transfer coefficients for the SVOC source and sink surfaces, respectively.

## Results and Discussion

### The Conditions for Neglecting the Sink Effect in Chambers

If a chamber is designed to neglect the sink effect, the time to reach steady state can be significantly shortened, and the analysis to determine C* (and consequently *y_0_*) can be simplified (equation (S13), Supporting Information S1), compared to when it cannot be neglected (equations (S5)-(S8), Supporting Information S1).

Taking the derivative of *y_0_* based on the definition of C* (C* = *C*/*y*_0_), we have
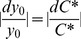
(14)


Equation (14) indicates that the influence of neglecting the sink effect on the determination of *y_0_* can be examined by analyzing the influence on C*. As a result, a relative error of C*, ε_C*_, is defined as the difference in gas-phase concentration between that obtained from equations (S5)-(S8) (*C*_1_*, with the sink effect) and that obtained from equation (S13) (*C*_2_*, without the sink effect), or,
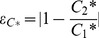
(15)


**Figure 2:**
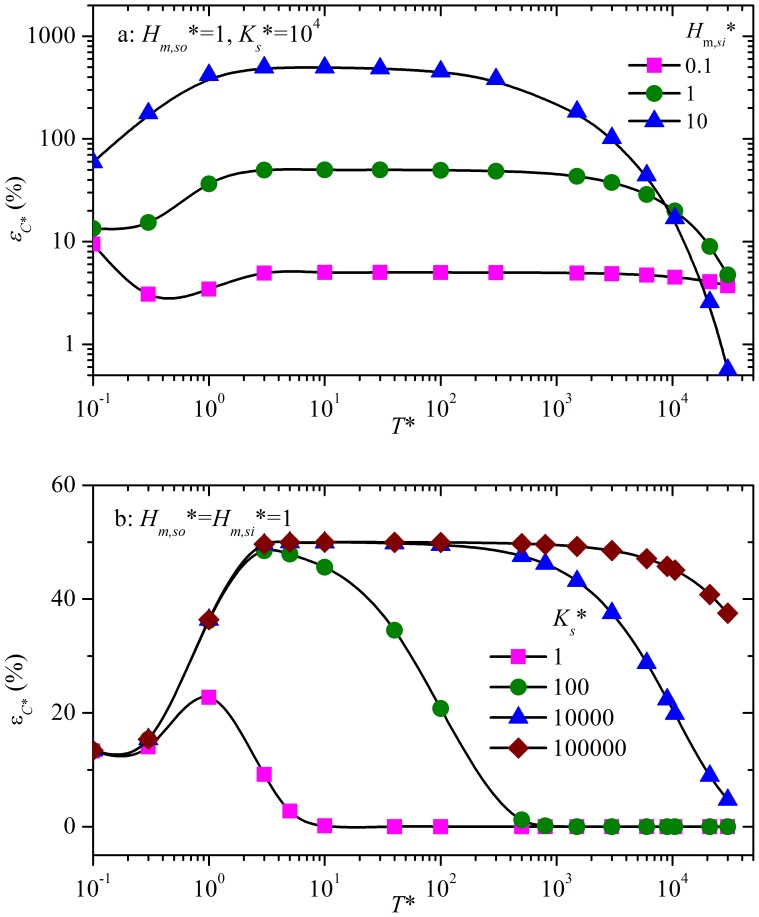
The influence of the sink effect on gas-phase SVOC concentration. (a) the mass transfer strength; (b) the sorption strength.

Based on the results of Liu et al. [27], the order of magnitude of the dimensionless parameters are: H_m,so_*∼10^0^, H_m,si_*∼10^0^, K_s_*∼10^4^. Figure 2 shows the influence of the sink effect on ε_C*_.

Figure 2 reveals that:

1. A strong mass transfer resistance at a sorption surface (e.g., H_m,si_* = 0.1), or a weak sorption surface (e.g., K_s_* = 1), both mean that the sink effect can be neglected. This is either because the strong mass transfer resistance keeps the SVOC from reaching the sorption surface, or because the surface has a weak affinity for the SVOC;
2. Sorption to the sink has little influence on C* in the initial stage, but when T*>1, the influence of the sink effect becomes significant. As the gas-phase concentration comes to steady state, the rate of SVOCs sorbing to a sink surface decreases, and the influence of the sink effect becomes less important as the sink approaches equilibrium with the gas-phase concentration;
3. For a specific scenario (with given values of H_m,so_*, H_m,si_* and K_s_*), there is a critical time, designated as T_c_*, after which the influence of the sink effect on C* can be neglected (ε_C*_ is smaller than 10%).

**Figure 3:**
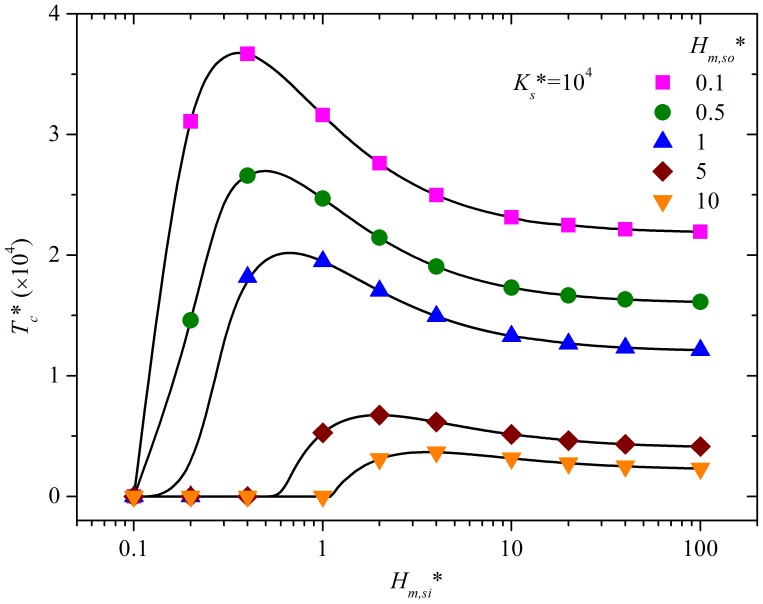
The relationship between T_c_* and H_m,si_* and H_m,so_* at K_s_* = 10^4^. As T_c_* is linearly proportional to K_s_* when K_s_* is higher than 0.1, it is easy to calculate T_c_* for other values of K_s_* using this figure.

Calculating ε_C*_ along with T* identifies the critical time (T_c_*) with ε_C*_ at that time and beyond being smaller than 10%. A convenient formula (equation (S16), Supporting Information S1) for T_c_* is obtained for the range of H_m,so_*, H_m,si_* and K_s_* of 0.1–10, 0.1–100 and 0–10^6^, respectively. A particular case of K_s_* = 10^4^ is illustrated in Figure 3.

Equation (S16) indicates that there are three simple and practical ways to design a chamber to neglect the sink effect from the beginning (i.e. T_c_* = 0): 1) increase source surface area (i.e. increase H_m,so_*); 2) decrease the sink surface area (i.e. decrease H_m,si_*); 3) use material with a lower sorptive capacity for chamber surfaces (i.e. decrease K_s_*). Other ways are to deliberately design the chamber shape and airflow pattern to decrease the mass transfer coefficient at sink surfaces (i.e. decrease H_m,si_*), and/or to increase the mass transfer coefficient at source surfaces (i.e. increase H_m,so_*). An additional practical solution is to increase the ventilation rate such that H_m,si_* is smaller than 0.11, meaning that H_m,si_* is smaller than H_m,so_* +0.11 (see equation (S16)).

### The Condition for Neglecting the Convective Mass Transfer at Sink Surfaces in Chambers

If a chamber is designed to neglect the mass transfer resistance at sorption surfaces, without neglecting the sink effect, the determination of C* (and consequently *y_0_*) can also be greatly simplified (equation (S15)) compared to when the mass transfer resistance cannot be neglected (equations (S5)-(S8)).

Similar to ε_C*_, ε_C,s*_ is defined as the difference in gas-phase concentration between that obtained from (S5)–(S8) (*C*_1_*, including convection resistance at sorption surfaces) and that obtained from (S15) (*C*_3_*, excluding convection resistance at sorption surfaces), or,
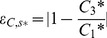
(16)


**Figure 4:**
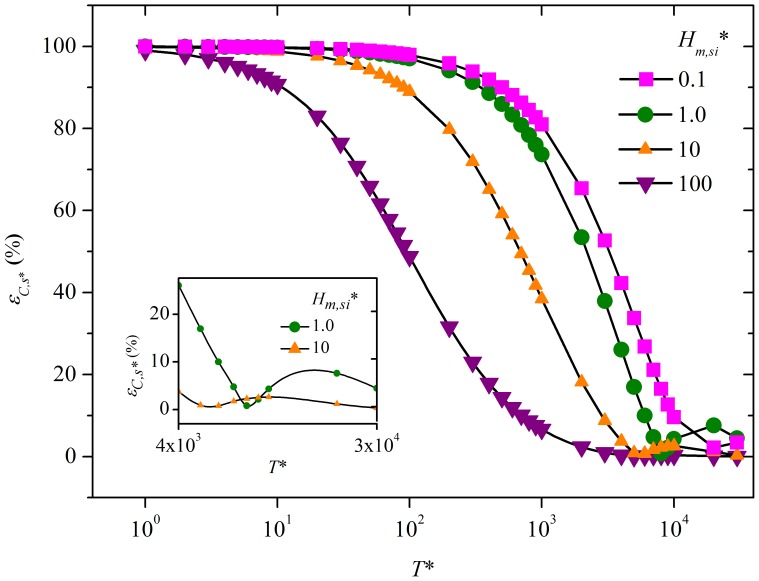
The influence of convective mass transfer at sink surfaces (*H_m_*_,si_*) on ε_C__*_. H_m,so_* = 1, K_s_* = 1.0×10^4^.

Figure 4 shows the influence of H_m,si_* on ε_C,s*_.

While T* increases, ε_C,s*_ would gradually decrease to zero in a general sense, but with a small increase of less than 10% for the case of H_m,si_* = 1.0 and 10 at T* of about 10^4^ (see the insert graph in Figure 4). For a specific scenario (with given values of H_m,so_*, H_m,si_* and K_s_*), there is a critical time, designated as T_c,s_*, after which the influence of convective mass transfer resistance at sink surface (H_m,si_*) on gas-phase SVOC concentration (C*) can be neglected (ε_C,s*_ is smaller than 10%).

**Figure 5:**
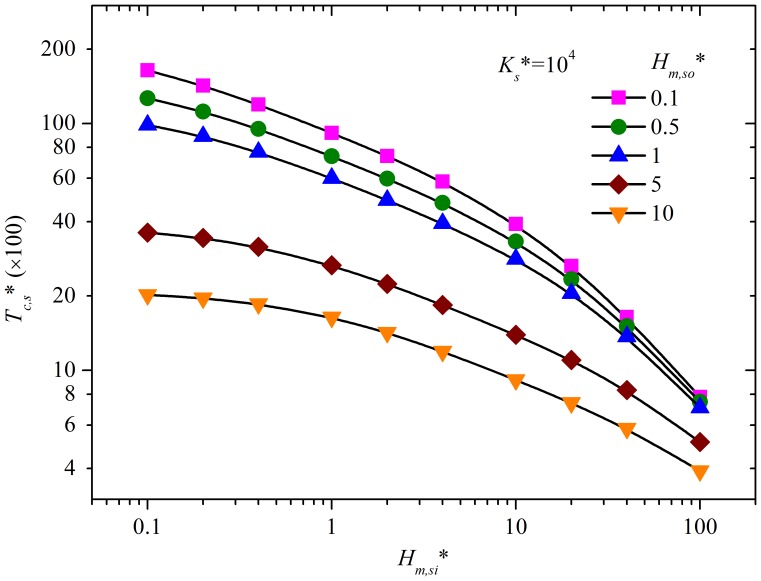
The relationship between T_c,s_* and H_m,si_* and H_m,so_* at K_s_* = 10^4^. As T_c,s_* is linearly proportional to K_s_*, it is easy to calculate T_c,s_* for other values of K_s_* using this figure.

Calculating ε_C,s*_ along with T* identifies the critical time (T_c,s_*) with ε_C,s*_ at that time and beyond being smaller than 10%. Results are shown in Figure 5, providing the condition for neglecting convective mass transfer resistance at sink surfaces. To make the determination of T_c,s_* convenient, a formula (equation (S17), Supporting Information S1) for T_c,s_* (ε_C,s*_ = 10%) is obtained for the range of H_m,so_*, H_m,si_* and K_s_* of 0.1–10, 0.1–100 and 10–10^6^, respectively.

Equation (S17) can be used to improve chamber design when the sink effect cannot be neglected. The practical methods presented in Section 4.1 are again applicable, except those for the mass transfer coefficient at sink surfaces and the ventilation rate. Increasing the mass transfer coefficient at sink surfaces is favored while the influence of ventilation rate is complicated and needs to be quantitatively evaluated by equation (S17).

### The Condition for Considering Material-phase SVOC Concentration as Constant in Chambers

As mentioned before, if a chamber is designed to be able to regard the material-phase SVOC concentration in the source as constant, the test can be greatly simplified. This has usually been the case in previous studies to investigate the emission mechanism of SVOCs [6], [16]. The dimensionless model presented here can help determine the general condition under which the assumption holds.

A dimensionless parameter, V_m_*, is introduced:
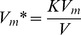
(17)where *V_m_* (m^3^) is the volume of SVOC source material and V_m_* is the dimensionless air volume needed to deplete the source material. Based on the previous results [16], [27] V_m_* is on the order of 10^10^–10^11^. If the emitted mass of SVOC is less than 10% of the initial mass before a chamber test ends, the material-phase SVOC concentration can be considered constant. A critical time, designated as T_c,m_*, therefore exists prior to which the material-phase SVOC concentration can be considered constant. For the following range of parameters: H_m,so_*: 10^0^–10^2^; H_m,si_*: 10^−1^–10^2^; K_s_*: 10^3^–10^7^, V_m_*: 10^9^–10^12^, a formula to determine T_c,m_* is obtained by correlating T_c,m_* with the other parameters:



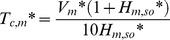
(18)Thus, neither H_m,si_* nor K_s_* has any influence on T_c,m_*. By the time of *T_c_*_,m_*, most of the emitted SVOC is not sorbed to sink surfaces, but has been exhausted from the chamber. Reducing the ventilation rate and increasing the surface area of the source are simple practical ways to lengthen the period during which the material-phase SVOC concentration can be considered constant. Equation (18) can help quantify practical ways to improve the chamber design.

## Illustrative Examples

**Figure 6:**
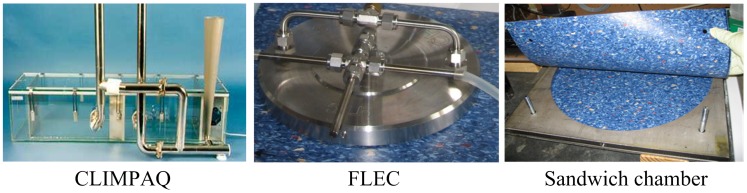
Photos of the three chambers.

To characterize exposure pathways and assess and control exposure and risk, accurate determination of *y_0_* is necessary. Based on the preceding analysis, an ideal chamber to determine source characteristic parameters is one which has no sink at all. But such an ideal chamber is not easy to achieve in practice. Currently, there are three chambers available in the literature to determine *y_0_*: CLIMPAQ [16], [27], FLEC [16] and the specially-designed or “sandwich” chamber [18], as shown in Figure 6. Detailed description of the experiment work has been provided previously [16], [18], and is only briefly reviewed here, with key information listed in Tables 1 and 2. Clean air is passed through the chambers with di-ethylhexyl phthalate (DEHP)-containing source material at room temperature. The air in the outlet is monitored with sorption tubes. The tubes are then thermally desorbed and analyzed with gas chromatography-mass spectroscopy to obtain the evolution of concentration as a function of time. The key parameters (e.g. *y_0_*) can be obtained by fitting the models to the evolving gas-phase concentration. In this section, the preceding analysis is illustrated by examining if the sink effect and the convective mass transfer resistance at sorption surfaces can be reasonably neglected for these three chamber studies, and if the material-phase SVOC concentration can be regarded as constant.

**Table 1:** Test conditions for the three chambers.

Chamber	*V* (L)	*A_e_* (m^2^)	*A_s_* (m^2^)	*Q* (/h)
CLIMPAQ	51	1.6	1.6	10
FLEC	0.035	0.018	0.018	7.7×10^2^
sandwich chamber	2.0	0.25	0.020	26

**Table 2:** The values of characteristic parameters and dimensionless parameters for the three chambers.

Chamber	*K_s_* (m)	*h_m,si_* (m/h)	*h_m,so_* (m/h)	H_m,si_*	H_m,so_*	K_s_*	V_m_* d
CLIMPAQ	2.1×10^3^	3.0	1.4^a^	9.0	4.3	6.5×10^4^	1.4×10^10^
FLEC^b^	8.5×10^3^	88	5.0^a^	59	3.4	4.4×10^6^	2.4×10^11^
sandwich chamber^b^	2.1×10^3^ c	61	1.7	24	8.6	2.1×10^4^	5.8×10^10^

a Estimated by Xu and Little [16];

b The values are the averaged results from fitting the duplicate data;

c Measured by Liu et al. [18];

d *C_m0_* is 2.6×10^11^ µg/m^3^ [16].

### The Sink Effect

**Figure 7:**
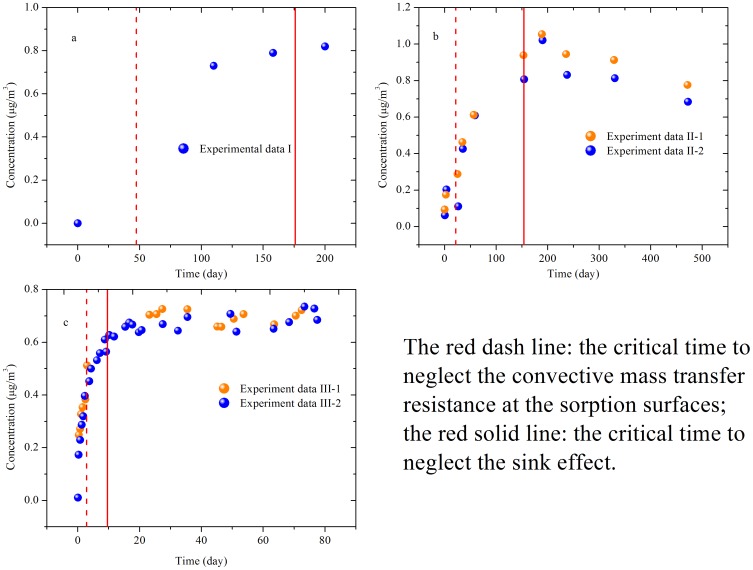
The distribution of experimental data compared to the critical time for the three chambers. (a) CLIMPAQ; (b) FLEC; (c) sandwich chamber.

The test conditions for the three chambers are listed in Table 1. The parameters involved can be obtained by fitting the experimental data with equations (6)-(9). Then the dimensionless parameters can be calculated, as shown in Table 2. Based on equation (S16), the critical dimensional time to neglect the sink effect can be determined, as shown by the red solid line in Figure 7. The experimental data obtained after the critical time is close to the steady-state concentration. Significant uncertainty will be introduced if all the data are used to determine the characteristic parameters by fitting with equation (11). The sink effect for these three chambers cannot be neglected when determining the characteristic parameters. Errors might be smaller if only the data obtained after the critical time are used, but it takes such a long time (see the red solid line in Figure 7) to reach the critical time that the cost of the chamber tests will be significantly increased.

### The Convective Mass Transfer Resistance at Sorption Surfaces

Using equation (S17) and values of parameters listed in Table 2, the critical dimensional time to neglect the convective mass transfer resistance at sorption surfaces for CLIMPAQ, FLEC and the sandwich chamber is determined and shown by the red dashed lines in Figure 7. As shown in Figure 7, most experimental data from each chamber was collected after the critical time, indicating that the condition of neglecting convective mass transfer resistance at sorption surfaces is satisfied for most data. Thus neglecting convective mass transfer resistance at sorption surfaces has insignificant influence on determining *y_0_*. We calculated *y_0_* by fitting the experimental data with equations (6)–(9) and equation (13), respectively, with results listed in Table 3 showing no significant difference, confirming the preceding analysis.

**Table 3:** Results of *y_0_* for the three chambers.

Chambers	*y_0_* (µg/m^3^)		Relative deviation (%)
	Including *h_m,si_*	Excluding *h_m,si_*	
CLIMPAQ	1.1	1.0	9.1
FLEC^a^	1.2	1.2	0
sandwich chamber^a^	0.78	0.72	7.7

a The values are the average of the results from fitting the duplicate data.

An effort to accurately determine *h_m,si_* is therefore not needed for these three chambers. In addition, equation (13) can be used to fit the experimental data in a much simpler way, compared to using equations (6)-(9). Furthermore, for the sandwich chamber, the condition of neglecting convective mass transfer resistance at sorption surfaces is satisfied much earlier than for the other two. Therefore, this chamber is more time-efficient. The effectiveness of the sandwich chamber can be explained by the fact that it has the smallest K_s_* and the highest H_m,so_*, which reduces the critical time to neglect the convection resistance, as shown in Figure 5.

### The Material-phase SVOC Concentration

The dimensional critical time, *t_c,m_*, before which the material-phase SVOC concentration can be treated as a constant, is determined by combining equation (18) and the definition of T*, with results listed in Table 4. The critical time is much longer than the duration of the three chamber runs, as indicated in Figure 7. Therefore, it is reasonable to assume that the material-phase DEHP concentration is constant in all three cases.

**Table 4:** The critical time to consider material-phase SVOC concentration as a constant in the three chambers.

Chambers	*t_c,m_* (day)
CLIMPAQ	7.1×10^6^
FLEC	1.8×10^6^
sandwich chamber	1.1×10^7^

## Conclusions

This work provided a dimensionless analysis of indoor SVOC source characteristics. Several practical and quantifiable ways to improve chamber design are identified. They can help to shorten the time needed for an experimental test and/or simplify the mathematical analysis of the experimental data. Specifically, it was found that:

1. The dimensionless gas-phase SVOC concentration in indoor air, C*, is a function of four dimensionless parameters - T* (the dimensionless time), K_s_* (the dimensionless sorption capacity of sink surfaces), H_m,so_* and H_m,si_* (the dimensionless mass transfer coefficients for the SVOC source and sink surfaces, respectively).
2. The applicable conditions of neglecting the sink effect and convective mass transfer resistance at sorption surfaces, and considering material-phase SVOC concentration as constant are: there is a critical time designated as T_c_*, such that when T*>T_c_*, the sink effect can be neglected; a critical time designated as T_c,s_*, such that when T*>T_c,s_*, the convective mass transfer resistance at sorption surfaces can be neglected; and a critical time designated as T_c,m_*, such that when T*<T_c,m_*, the material-phase SVOC concentration can be considered constant. Equations (S16), (S17) and (18) can be used to determine T_c_*, T_c,s_* and T_c,m_*, respectively.
3. Several simple practical ways have been proposed to either neglect the sink effect, or the convection at sorption surfaces, or consider the material-phase SVOC concentration as constant. Increasing the surface area of the source is the common solution for all three purposes. One advantage of the present study is that the effect of the practical ways to improve chamber design can be quantified using equations (S16), (S17) and (18).
4. Three different chambers used to examine SVOC source characteristics, CLIMPAQ, FLEC and the specially-designed chamber, were evaluated based on the preceding analysis. It was found that for these three methods, the sink effect cannot be neglected, while neglecting convective mass transfer resistance at sorption surfaces and considering the material-phase concentration as constant are reasonable to determine *y_0_*. Considering the time to satisfy the condition, the specially-designed chamber method is more time-efficient (2.9 days) than FLEC (22 days) and CLIMPAQ (47 days). This is due to the fact that the method has the smallest K_s_* and the highest H_m,so_*.

## Supporting Information

Supporting Information S1 A: The dimensionless model development (equations (S1)–(S15)). B: The dimensionless correlations for T_c_* and T_c,s_* (equations (S16) and (S17)).
(DOC)
