# Supplementary material for: Convenient, Rapid and Accurate Measurement of SVOC Emission Characteristics in Experimental Chambers
Source: PLoS One. 2013 Aug 28;8(8):e72445. doi: 10.1371/journal.pone.0072445 (PMC3756072; doi:10.1371/journal.pone.0072445)
Supplement: Supporting Information S1 — A: The dimensionless model development (equations (S1)–(S15)). B: The dimensionless correlations for Tc* and Tc,s* (equations (S16) and (S17)). (DOC) [file pone.0072445.s001.doc]

**The Supporting Information (SI)**

**A: The dimensionless model development**

**B: The dimensionless correlations for *T_c_** and *T_c,s_****

**SI-A. The dimensionless model development**

With the definition of the dimensionless parameters, equations (1), (3) and (4) can be rewritten as:

| $E*=H_{m,so}*\left(1-C*\right)$ | (S1) |
| --- | --- |
| $S*=H_{m,si}*\left(C*-C_{s}*\right)=K_{s}*\frac{dC_{s}*}{dT*}$ | (S2) |
| $\frac{dC*}{dT*}=E*-C*-S*$ | (S3) |

The initial conditions are:

| $C*\left(T*=0\right)=C_{s}*\left(T*=0\right)=0$ | (S4) |
| --- | --- |

The analytical solution to equations (S3) - (S4) is

| $C*=X*e^{a*T*}+Y*e^{b*T*}-\left(X*+Y*\right)$ | (S5) |
| --- | --- |

and *X** and *Y** are as:

| $X*=\frac{H_{m,so}*\alpha*}{a*}$, $Y*=\frac{H_{m,so}*\beta*}{b*}$ | (S6) |
| --- | --- |

where *α** and *β** are the roots of the following set of equations:

| $\left\{\begin{matrix} \alpha*+\beta*=1 \\ \alpha*b*+\beta*a*=-\frac{H_{m,si}*}{K_{s}*} \end{matrix}\right.$, | (S7) |
| --- | --- |

and *a** and *b** (*a** > *b**) are the roots of the following equation:

| $\lambda^{2}+\left(\frac{H_{m,si}*}{K_{s}*}+H_{m,so}*+1+H_{m,si}*\right)\lambda+\frac{H_{m,si}*}{K_{s}*}\left(H_{m,so}*+1\right)=0$ | (S8) |
| --- | --- |

The dimensionless SVOC concentration, *C**, the dimensionless emission rate, *E**, and the dimensionless sorption rate, *S**, take the following functional forms:

| $C*=f_{1}\left(H_{m,so}*,H_{m,si}*,K_{s}*,T*\right)$ | (S9) |
| --- | --- |
| $E*=H_{m,so}*\left(1-C*\right)=f_{2}\left(H_{m,so}*,H_{m,si}*,K_{s}*,T*\right)$ | (S10) |
| $S*=H_{m,si}*\left(C*-C_{s}*\right)=f_{3}\left(H_{m,so}*,H_{m,si}*,K_{s}*,T*\right)$ | (S11) |

When the sink effect is neglected, equation (S2) simplifies to:

| $S*=0$ | (S12) |
| --- | --- |

Then the analytical solution to equations (S1), (S3), (S4) and (S12) is

| $C*=\frac{H_{m,so}*}{H_{m,so}*+1}\left(1-e^{-\left(1+H_{m,so}*\right)T*}\right)$ | (S13) |
| --- | --- |

When the convective mass transfer resistance at sorption surfaces is neglected (i.e., *H_m,si_** is regarded as infinite), equation (S2) simplifies to:

| $C_{s}*=C*$ | (S14) |
| --- | --- |

Then the analytical solution to equations (S1), (S3), (S4) and (S14) is

| $C*=\frac{H_{m,so}*}{H_{m,so}*+1}\left(1-e^{-\frac{1+H_{m,so}*}{1+K_{s}*}T*}\right)$ | (S15) |
| --- | --- |

**SI-B. The dimensionless correlations**

| $T_{c}*=\left\{\begin{matrix} 0 H_{m,si}*<f_{1} or K_{s}*<0.1 \\ K_{s}*\left[p_{1}\left(\ln H_{m,si}*\right)^{2}+p_{2}\ln H_{m,si}*+p_{3}\right] f_{1}<H_{m,si}*<f_{2} and K_{s}*>0.1 \\ K_{s}*\left[p_{4}\left(\ln H_{m,si}*\right)^{2}+p_{5}\ln H_{m,si}*+p_{6}\right] f_{2}<H_{m,si}*<100 and K_{s}*>0.1 \end{matrix}\right.$ $f_{1}=0.098H_{m,so}*+0.11$, $f_{2}=0.32H_{m,so}*+0.36$ | (S16) |
| --- | --- |

where $p_{1}=0.082\left(\ln H_{m,so}*\right)^{2}+0.34\ln H_{m,so}*-1.5$, $p_{2}=-0.19\left(\ln H_{m,so}*\right)^{3}+0.015\left(\ln H_{m,so}*\right)^{2}+1.9\ln H_{m,so}*-1.5$, $p_{3}=-0.11\left(\ln H_{m,so}*\right)^{2}-0.40\ln H_{m,so}*+1.5$, $p_{4}=-0.0018\left(\ln H_{m,so}*\right)^{2}-0.016\ln H_{m,so}*+0.051$, $p_{5}=0.014\left(\ln H_{m,so}*\right)^{2}+0.11\ln H_{m,so}*-0.38$, $p_{6}=-0.034\left(\ln H_{m,so}*\right)^{2}-0.60\ln H_{m,so}*+1.9$.

| $T_{c,s}*=\frac{K_{s}*}{10^{2}}\left[p_{s1}\left(\ln H_{m,si}*\right)^{2}+p_{s2}\ln H_{m,si}*+p_{s3}\right]$ | (S17) |
| --- | --- |

where $p_{s1}=0.0069\left(\ln H_{m,so}*\right)^{2}-0.45\ln H_{m,so}*+0.96$, $p_{s2}=0.21\left(\ln H_{m,so}*\right)^{2}+5.7\ln H_{m,so}*-16$, $p_{s3}=-1.6\left(\ln H_{m,so}*\right)^{2}-17\ln H_{m,so}*+61$.
